# Gamification for health promotion: systematic review of behaviour change techniques in smartphone apps

**DOI:** 10.1136/bmjopen-2016-012447

**Published:** 2016-10-04

**Authors:** E A Edwards, J Lumsden, C Rivas, L Steed, L A Edwards, A Thiyagarajan, R Sohanpal, H Caton, C J Griffiths, M R Munafò, S Taylor, R T Walton

**Affiliations:** 1 Centre for Primary Care and Public Health, Bart's and The London School of Medicine and Dentistry, Queen Mary University of London, London, UK; 2 School of Experimental Psychology, University of Bristol, Bristol, UK; 3 MRC Integrative Epidemiology Unit at the University of Bristol, Bristol, UK; 4 Faculty of Health Sciences, University of Southampton, Southampton, UK; 5 Institute of Liver Studies, King's College Hospital, London, UK; 6 Department of Computing and Information Systems, Kingston University, London, UK

**Keywords:** PUBLIC HEALTH

## Abstract

**Objective:**

Smartphone games that aim to alter health behaviours are common, but there is uncertainty about how to achieve this. We systematically reviewed health apps containing gaming elements analysing their embedded behaviour change techniques.

**Methods:**

Two trained researchers independently coded apps for behaviour change techniques using a standard taxonomy. We explored associations with user ratings and price.

**Data sources:**

We screened the National Health Service (NHS) Health Apps Library and all top-rated medical, health and wellness and health and fitness apps (defined by Apple and Google Play stores based on revenue and downloads). We included free and paid English language apps using ‘gamification’ (rewards, prizes, avatars, badges, leaderboards, competitions, levelling-up or health-related challenges). We excluded apps targeting health professionals.

**Results:**

64 of 1680 (4%) health apps included gamification and met inclusion criteria; only 3 of these were in the NHS Library. Behaviour change categories used were: feedback and monitoring (n=60, 94% of apps), reward and threat (n=52, 81%), and goals and planning (n=52, 81%). Individual techniques were: self-monitoring of behaviour (n=55, 86%), non-specific reward (n=49, 82%), social support unspecified (n=48, 75%), non-specific incentive (n=49, 82%) and focus on past success (n=47, 73%). Median number of techniques per app was 14 (range: 5–22). Common combinations were: goal setting, self-monitoring, non-specific reward and non-specific incentive (n=35, 55%); goal setting, self-monitoring and focus on past success (n=33, 52%). There was no correlation between number of techniques and user ratings (p=0.07; r_s_=0.23) or price (p=0.45; r_s_=0.10).

**Conclusions:**

Few health apps currently employ gamification and there is a wide variation in the use of behaviour change techniques, which may limit potential to improve health outcomes. We found no correlation between user rating (a possible proxy for health benefits) and game content or price. Further research is required to evaluate effective behaviour change techniques and to assess clinical outcomes.

**Trial registration number:**

CRD42015029841.

**Strengths and limitations of this study**

- This is the first comprehensive systematic review examining the use of behaviour change techniques in smartphone games aimed at changing health-related behaviours.
- We rigorously evaluated behaviour change techniques and classified them using the Behaviour Change Technique Taxonomy v1.
- We identify individual behaviour change techniques and combinations of techniques commonly used in smartphone games to facilitate development of more effective applications in future.
- We screened only 1680 top-rated apps in the most popular app stores; so while our sample may be representative of apps in common use, we did not examine the full repertoire of apps offered by developers.
- We were not able to assess the clinical benefits or potential harms from using the apps since none have been rigorously evaluated.

## Introduction

Smartphone use has increased rapidly in recent years in developed and developing countries. There are over 2 billion smartphone users globally in 2016 and by 2018 one-third of the world's population will use smartphones.^1^ China had 500 million smartphone users in 2014, and in 2016, India will exceed 200 million users overtaking the USA as the world's second-largest smartphone market.^1^

Accompanying this rapid growth in smartphone use is a huge expansion in applications targeting health and health-related behaviours. Over 100 000 health applications (apps) are available worldwide for smartphones with exercise, diet and weight management apps being the most popular downloads.^2–4^ Consumers are keen to access health information on their mobile devices and >500 million people globally currently use mobile health applications.^5^ However, most health applications for smartphones have very simple functions and do little more than provide basic information.^6^ There is little evidence that public health practitioners and users participate in the design of health apps and most apps do not contain theoretically consistent behaviour change techniques.^7–18^ Very few apps comply with regulatory processes or have had their effectiveness formally assessed,^6^ ^8^ ^16^ ^19^ leading to concerns about lack of benefit or even potentially harmful apps.^19^

While there is guidance from Apple and Android stores on criteria that must be met for app inclusion,^20^ ^21^ this focuses on ensuring that app content is not of a violent, illegal or sexual nature, that it functions reliably and that intellectual property is secured. The National Health Service (NHS) Health Apps Library uses a more rigorous approach with a clinical assurance team to ensure apps comply with trusted sources of information and to identify apps that may potentially cause harm.^22^ However, currently, there is no requirement to demonstrate effectiveness in modifying either behavioural or clinical outcomes or that the app complies with regulatory frameworks (http://www.fda.gov/MedicalDevices/DigitalHealth/MobileMedicalApplications/default.htm, https://www.gov.uk/government/publications/medical-devices-software-applications-apps).

In parallel with the growth in health apps, there has been a remarkable increase in gaming on personal computers, dedicated game consoles and on smartphones. Games now form the largest market share of apps comprising 33% of all downloads.^23^ It is estimated that 69% of people in the UK aged 8–74 are playing games on average 14 hours per week.^24^ Of these players, 52% are female and the average age is 31 years. ‘Gamification’ harnesses a desire for competition, incorporating ‘gaming elements’ such as badges, leaderboards, competitions, rewards and avatars to engage and to motivate people.^25^ The use of gamification is increasingly popular for training programmes in industry with a projected $2.8 billion spend on gamification by businesses in 2016.^26^ Higher education institutions have also integrated gaming techniques into their teaching programmes.^27^

While there are successful health applications of gamification on Super Nintendo, Nintendo Wii and personal computers, gamification in mobile health is, perhaps surprisingly, a relatively new concept.^28–31^ Gamification can be effective in promoting and sustaining healthy behaviours, tapping into playful and goal-driven aspects of human nature. Gamification strategies such as goal setting, providing feedback on performance, reinforcement, comparing progress and social connectivity share key elements with established health behaviour change techniques.^32^

A behaviour change technique is ‘an observable, replicable and irreducible component of an intervention designed to alter or redirect causal processes that regulate behaviour; that is, a technique is proposed to be an “active ingredient” (e.g., feedback, self-monitoring, reinforcement)’.^7^ These techniques have been clearly defined, linked with theories of behaviour change and classified into an internationally recognised taxonomy, comprising 93 individual techniques, grouped into 16 behaviour change categories.^7^

This taxonomy builds on previous work to identify the active components of complex interventions.^8^ ^33–37^ For example, Dombrowski *et al* coded behaviour change techniques for obese adults with obesity-related comorbidities in behavioural interventions applying a 26-category taxonomy developed by Abraham *et al*.^34^ ^38^

Although apps have proliferated, work aiming to characterise the use of behaviour change techniques in smartphone apps and smartphone games is relatively novel. Two reviews include Direito *et al*, who used a 26-category taxonomy developed by Abraham *et al*,^38^ ^39^ and Conroy *et al*, who used the Coventry, Aberdeen and London-Revised (CALO-RE) developed also by Michie *et al* and found the limited use of behaviour change techniques in diet and physical activity apps.^8^ ^40^ Crane *et al*^41^ examined the use of behaviour change techniques in alcohol reduction apps using the BCT taxonomy (v1). Findings again found the limited use of behaviour change techniques.

Here we provide the first comprehensive systematic review of behaviour change techniques in smartphone games classified using the BCT taxonomy (v1) developed by Michie *et al* comprising 16 behaviour change categories and 93 individual techniques. The purpose of this review is to identify appropriate behaviour change techniques and combinations of techniques for use in this setting to facilitate development of more effective smartphone games to promote health.^7^

## Methods

We identified all English language health apps for all ages (free and for purchase) that incorporated gamification. We defined gamification as use of at least one of the following techniques: rewards, prizes, avatars, badges, leaderboards, competitions and health-related challenges. We searched the official Apple and Android app stores (https://play.google.com/store, https://itunes.apple.com) and selected ‘top-rated’ apps as defined by the store. The rating is derived from number of downloads and daily revenue generated.^42^ We also searched the NHS Health Apps Library (https://apps.nhs.uk). The protocol for this review has been published and is available as online supplementary prospero file. Prospero registration number: CRD42015029841.

10.1136/bmjopen-2016-012447.supp1 supplementary prospero file

### Search strategy

The initial search was conducted by one review author (EAE) from 1 April 2014 to 30 June 2015 examining all apps in the ‘top-rated’ categories in each app store. Data from apps meeting inclusion criteria were recorded in a prepiloted, standardised, structured data collection form.

### Inclusion/exclusion criteria

Inclusion criteria were broad, aiming to identify all ‘top-rated’ smartphone apps incorporating gaming elements, which were marketed to the general public (table 1).

**Table 1:** Inclusion and exclusion criteria

Inclusion criteria	Exclusion criteria
English language smartphone apps	Apps designed for tablet computers
Apps available through Google play and iTunes or NHS app store	Non-English language apps
Apps included in the medical, health and wellness or health and fitness section of Google play and iTunes and all NHS apps	Apps in other sections of the stores
Apps including gamification techniques: rewards, prizes, avatars, badges, leaderboards, competitions, health-related challenges	Smartphone apps that do not contain gamification techniques
Smartphone apps targeted at users of any age	Smartphone apps designed for healthcare professionals
Free and paid smartphone apps	Apps not targeting to change a physical health behaviour
Apps targeting to change a physical health behaviour	Apps that did not have customer ratings available

Inclusion and exclusion criteria that were established for the initial search of the official Apple, Android app stores and NHS Health Apps Library aiming to identify all ‘top-rated’ smartphone apps incorporating gaming elements, which were marketed to the general public.

NHS, National Health Service.

### Coding the apps for behaviour change techniques

All apps meeting inclusion criteria were downloaded onto test devices. The same make and model of test device was used throughout the evaluation (LG Nexus 5 Android or iPhone 5c). Test devices were unmodified consumer-grade smartphones running up-to-date versions of their mobile operating system. The same version of each app was used throughout testing. The entire app content was coded for behaviour change techniques, including text, images, video and other multimedia content. Apps found in the Apple store and Google Play store were not included twice and were recorded only in the Apple iPhone data.

Two researchers trained in behaviour change technique coding (EAE and JL) coded apps independently. App content was coded using the BCT taxonomy (v1).^7^ Techniques were classified as either present or absent. An example of the coding process and application of behaviour change techniques to app content is provided (see online supplementary figure S1). The number of individual behaviour change techniques included in each app was counted. There was no count of the frequency in which techniques were used in each individual app.

10.1136/bmjopen-2016-012447.supp2 supplementary figures

We used Cohen's κ to assess inter-rater reliability of BCT coding at the initial stage of review. There was substantial agreement between the two reviewers (κ=0.79, 95% CI 0.76 to 0.81). All discrepancies in reviewer coding were then resolved through discussion with a third trained reviewer (LS), a health psychologist.

Codes from each reviewer were recorded on a standardised, structured form. We recorded information on app version, date of first release, date of latest update, publisher, description, main function, target user, special features and number of downloads where available. Missing data were requested from the author/publisher of the app or from the Apple/Android stores.

### Synthesis of results

A qualitative and quantitative synthesis was conducted with calculation of basic descriptive statistics. Behaviour change technique use, including categories, individual techniques and combinations of techniques, was analysed. Comparison was made between the number of behaviour change techniques included, user rating and price. Correlations were determined using Spearman's rank correlation coefficient (r_s_), calculated with GraphPad Prism V.6.

## Results

**Figure 1:**
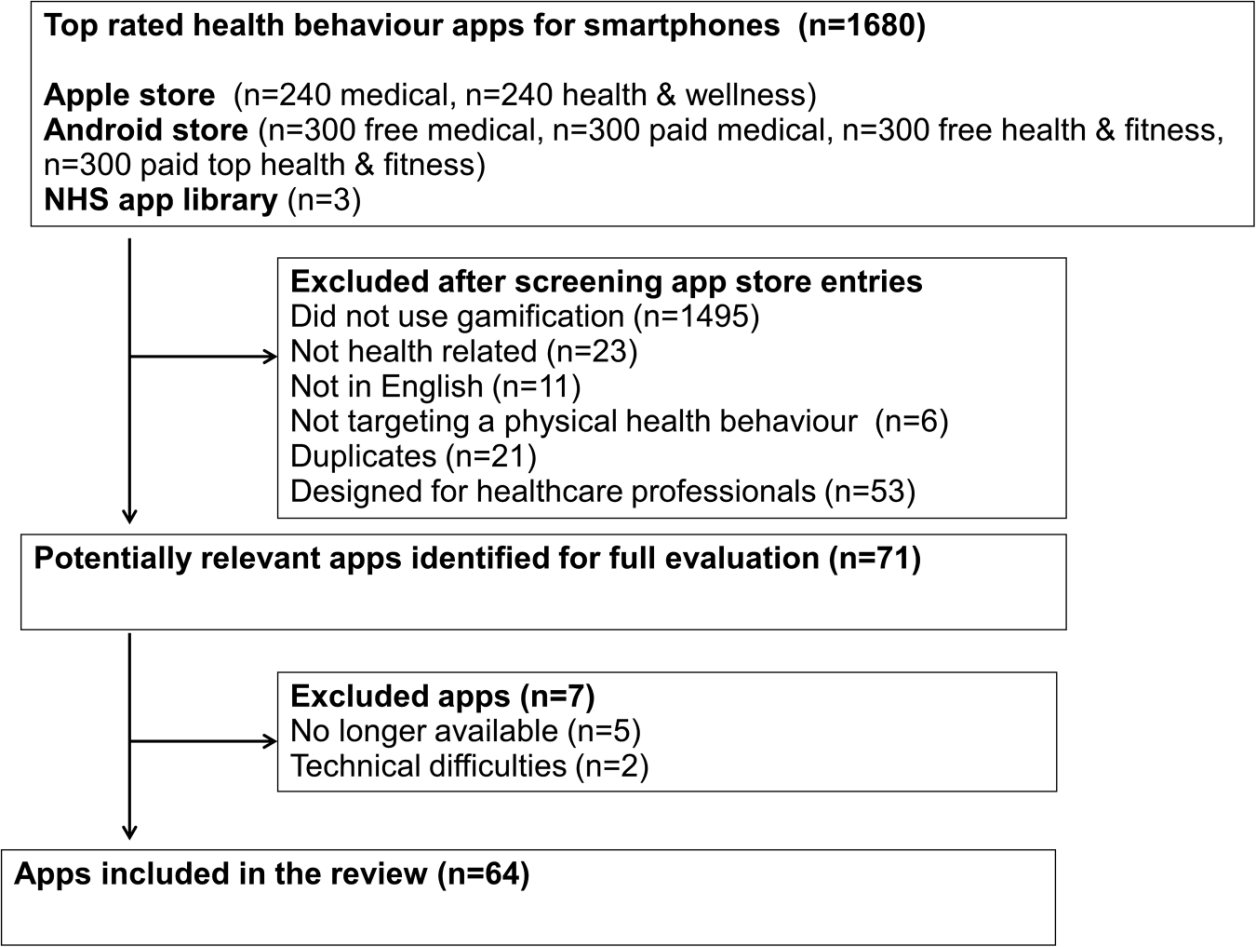
Flow chart of the app selection process, including total number of apps screened, number of apps that met inclusion criteria, number of apps that were included in the review and total number of apps that were excluded. NHS, National Health Service.

We screened 1680 medical, health and wellness or health and fitness apps of which 64 (4%) met inclusion criteria (figure 1). Although the initial search was conducted by one review author (EAE), the inclusion and exclusion criteria were defined a priori and agreed by three authors JL, LS and RTW. Additional discussions occurred during this initial search period between EAE and other review authors about inclusion of particular apps.

Apple displays 240 top-rated medical and 240 health and wellness apps comprising free and paid apps. Android displays free and paid apps separately, displaying their 300 top-rated free medical apps, 300 top-rated paid medical apps, 300 top-rated free health and fitness and 300 top-rated paid health and fitness apps. Thus, more Android than Apple apps were included.

In the apps meeting inclusion criteria, targeted behaviour changes included increasing/improving exercise (n=45, 70%), improving fitness (n=11, 17%), smoking cessation (n=4, 6%), encouraging oral hygiene (n=2, 3%), weight loss (n=1, 2%) and blood glucose measurement adherence (n=1, 2%, see online supplementary table S1).

10.1136/bmjopen-2016-012447.supp3 supplementary table Characteristics of included apps

**Figure 2:**
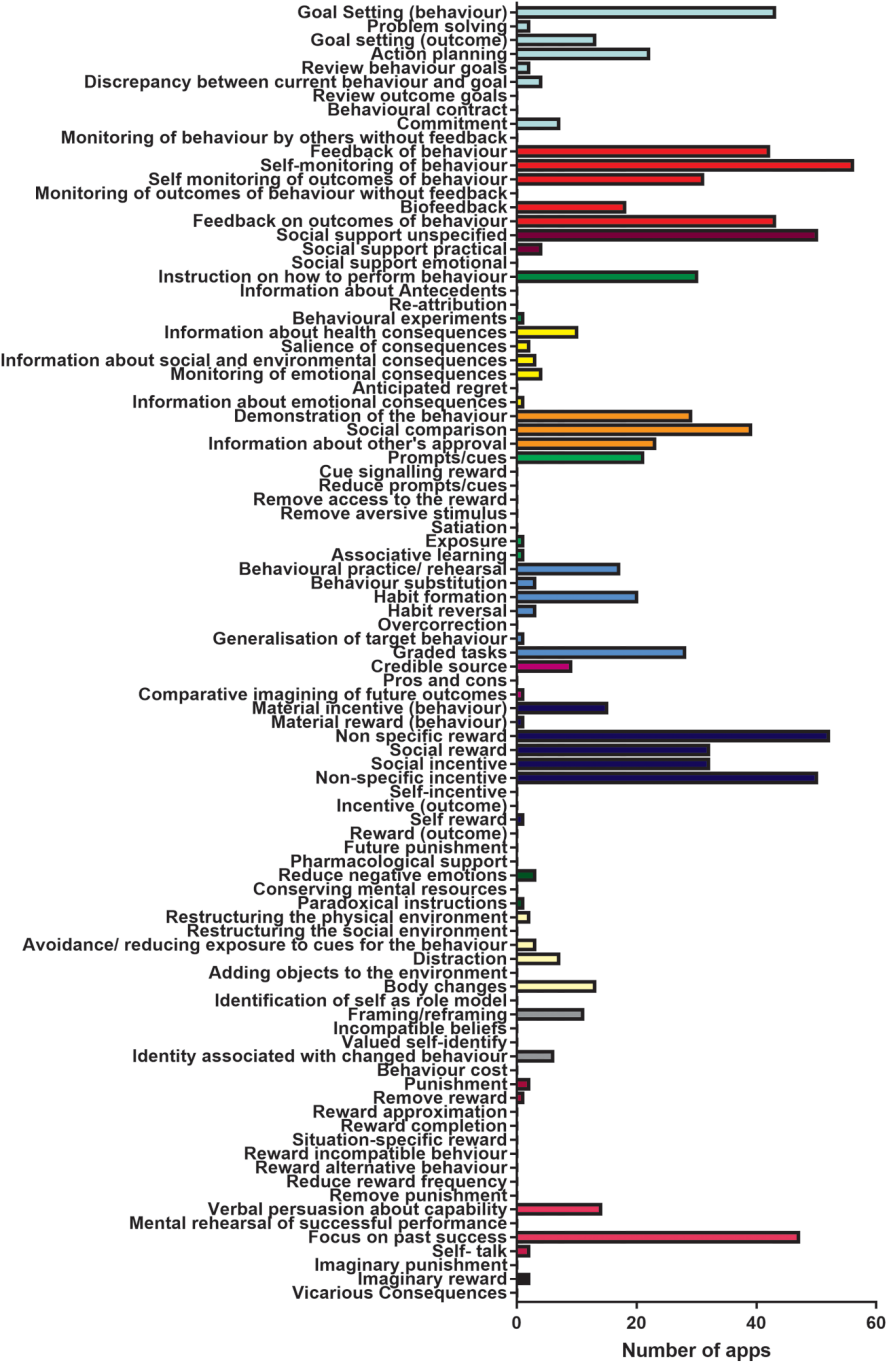
Number of apps to use the individual 93 behaviour change techniques as derived from a standard taxonomy of behaviour change techniques used in health behaviour change research.^7^

The median number of behaviour change techniques was 14 (range: 5–22) with a negatively skewed distribution (see online supplementary figure S2). The most common behaviour change categories were: feedback and monitoring (n=60, 94% of apps), comparison of behaviour (n=52, 81% of apps), and reward and threat (n=52, 81% apps). The most used individual techniques were: self-monitoring of behaviour (n=55, 86% apps), non-specific reward (n=49, 82% apps), non-specific incentive (n=49, 82% apps), social support unspecified (n=48, 75% apps) and focus on past success (n=47, 73% of apps; table 2; figure 2).

**Table 2:** Behaviour change technique categories included in apps

BCT taxonomy category groupings	Number of apps to use category	%
Feedback and monitoring	60	94
Comparison of behaviour	52	81
Reward and threat	52	81
Self-belief	51	80
Repetition and substitution	50	78
Social support	48	75
Goals and planning	46	72
Shaping knowledge	25	39
Associations	20	31
Antecedents	18	28
Identity	12	19
Natural consequences	9	14
Comparison of outcomes	5	8
Regulation	1	2
Scheduled consequences	3	5
Covert learning	2	3

Number and percentage of apps to use the 16 behaviour change techniques as derived from a standard taxonomy of behaviour change techniques used in health behaviour change research.^19^

BCT, behaviour change technique.

Forty-two of 93 (45%) behaviour change techniques in the taxonomy were not used in any app.

Frequently used combinations of techniques were based on self-monitoring and goal setting with the addition of either focus on past success (n=33, 47%) or non-specific rewards and incentives (n=33, 47%; table 3).

**Table 3:** Common combinations of behaviour change techniques

Technique combination	Number of apps to use combination, N (%)
Goal setting, self-monitoring, non-specific reward, non-specific incentive	35 (55)
Goal setting, self-monitoring, focus on past success	33 (51)
Goal setting, self-monitoring, non-specific reward, non-specific incentive, focus on past success	31 (48)
Goal setting, self-monitoring, feedback of behaviour, social support unspecified, focus of past success	27 (42)
Goal setting, feedback of behaviour, self-monitoring	28 (44)
Goal setting, feedback of behaviour, self-monitoring, social support unspecified, non-specific reward, non-specific incentive, focus past success	26 (41)
Goal setting, feedback of behaviour, self-monitoring, feedback of outcome of behaviour, social support unspecified, non-specific reward, non-specific incentive, focus on past success	22 (34)

Number and percentage of apps to use commonly identified combinations of behaviour change techniques.

Median user rating was 4.5 (range: 2.5–5). There was no correlation between the number of behaviour change techniques and customer ratings (p=0.07; r_s_=0.23).

Twenty-three apps (36%) were available to purchase and the remainder were free. The median cost of the paid apps was £1.99 (range: £0.62–£3.10). There was no correlation between number of behaviour change techniques and price (p=0.45; r_s_=0.10).

Only three apps were included in the NHS Health Apps Library: Change 4 Life fun generator by NHS choices, Zombies Run! and Zombies Run! 5k Training.

## Discussion

### Main findings

Despite a rapid increase in the use of gamification in the commercial and education sectors, smartphone applications using gamification for promoting health are currently limited. Our review highlights wide variation in the use of behaviour change techniques; however, all apps reviewed included at least five recognised behaviour change techniques, most commonly feedback and monitoring, comparison of behaviour, and reward and threat. It is also encouraging that app developers are using combinations of behaviour change techniques which are theoretically consistent such as goal setting, self-monitoring and non-specific reward.

### Results in the context of other studies

We found that self-regulatory behaviour change techniques were most commonly used (feedback and monitoring including self-monitoring of behaviour). These techniques are also commonly used in non-gamified apps targeting physical activity, healthy eating and alcohol reduction.^39^ ^40^ ^41^ The effectiveness of these techniques in achieving behaviour change is supported by findings from a wide range of studies^8^ ^33–37^ and linked to control theory.^37^ Control theory suggests that setting goals, monitoring of behaviour, receiving feedback and reviewing relevant goals in the light of feedback may be effective in changing behaviour^43^ and is one of a broader group of theories involving feedback loops and self-regulation.^44^

Frequently used behaviour change categories were comparison of behaviour and reward and threat. Common individual behaviour change techniques were social support unspecified, non-specific reward, non-specific incentive and focus on past success. We suggest that the use of some of these techniques may be driven by ease of implementation in smartphone games with an internet connection. Sharing activity on social media is a common feature of mobile apps and is easy to integrate into app design. Social support as a behaviour change technique is also common in physical activity apps.^40^

Other reviews have found that the behaviour change technique providing instruction on how to perform behaviour has featured highly among physical activity apps (n=33, 83% of apps)^39^ (n=111, 66% of apps);^40^ however, this technique was found in relatively few apps in our review (n=25, 39% of apps). It is possible that this technique may be more suited to physical activity apps since it was not found in apps to reduce alcohol consumption.^41^ Alcohol reduction apps also featured a range of techniques not found in smartphone games: facilitate self-recording, provide information on consequences, give options for additional and later support, and offer/direct towards appropriate written materials.^41^ While these techniques may be more suited to alcohol reduction apps, it is also possible that they do not lend themselves to use on the gaming platform.

One previous meta-analysis examined combinations of health behaviour change techniques using classification and regression trees and suggested that provide information about behaviour and prompt intention formation was one of the most effective combinations;^45^ however, comparison with our findings is problematic because the study used the earlier 26-category taxonomy^38^ which does not easily translate into the more recent 93 category taxonomy (v1).^7^

A second meta-analysis of internet-based interventions suggested that number of techniques included in the intervention and the resulting behaviour change outcomes were directly related.^46^ This review also suggested benefit from linking techniques to behaviour change theory. We were not able to examine effects on outcomes because of lack of outcome data, although we saw no relation between behaviour change technique content and user rating which may be a proxy for outcome. Several studies in other clinical settings find no relationship between number of behaviour change techniques and health outcome, for example, in obesity, healthy eating and physical activity,^34^ ^35^ ^37^ although these studies did not specifically examine effects using a technology-based delivery method. One study examining technology-based delivery found that popularity and user ratings were only weakly associated with behaviour change technique content.^41^

We found a high number of behaviour change techniques in each smartphone game (median: 14, range: 5–22). This figure is higher than previous reviews of non-app interventions to promote healthy eating (mean: 6, range: 1–13)^38^ and physical activity (mean: 6, range: 1–13)^38^ (mean: 6, SD: 3.1)^37^ (mean: 8, range: 2–18).^39^ Two other reviews of behaviour change techniques in physical activity and non-gamified alcohol reduction apps found a slightly lower number (mean: 4.2, range: 1–13)^40^ (mean: 3.6, range: 0–13).^41^ This may be related to the overlap between gamification methodology and health behaviour change techniques.

While there was no overall relationship between user rating and behaviour change technique content, one particular app deserves mention. ‘Diabetes Companion’ by mySugr has a 5/5* customer rating in the app store and used 18 behaviour change techniques. The Diabetes Companion is a charming, sometimes outspoken, diabetes monster that aims to make diabetes monitoring and data collection useful and fun in everyday life. The app is approved as a medical device by the Food and Drug Administration in the USA and has a Conformité Européene (CE) mark. Elements of gamification in the app and immediate feedback help to keep players motivated and involved in self-management. While there is no evaluation against health outcomes, this app may nevertheless provide a model for employing gamification and health behaviour techniques in smartphone apps.^47^ ^48^

We found that the price of an app was unrelated to number of behaviour change techniques reinforcing a similar finding from a content analysis of exercise apps.^49^ However, other earlier studies showed a positive relationship between price and behaviour change technique content.^14^ ^39^ ^50^ The disparity between findings could be explained by the recent rise in Freemium apps, which are free to download, but then apply charges for additional features.^51^

### Strengths and weaknesses

This is the first comprehensive review of the use of behaviour change techniques in smartphone games using the most recent behaviour change taxonomy.^7^ One previous review found limited use of behaviour change theory in gamified health apps.^3^ The review focused only on free physical activity and diet apps in the Apple store and used 13 core health behaviour constructs rather than a standard taxonomy of behaviour change techniques. Another review used the BCT taxonomy (v1), however, considered only non-gamified alcohol reduction apps.^41^

A further strength of this review is that we considered combinations of behaviour change techniques that were used in the apps. Many of the existing reviews report individual behaviour change techniques rather than combinations. However, our aim was only to identify the combinations of techniques that smartphone game developers are currently using. We had insufficient power to examine effects of theoretically consistent groups of techniques on proxy outcomes such as user rating or price. This is an interesting area of work requiring further research in larger databases, which would ideally include behavioural and clinical outcomes.^52^

While there may be a degree of subjectivity when coding behaviour change techniques using taxonomies,^53^ this would have been reduced by independent coding by two trained researchers.^53^ In addition, we demonstrated substantial agreement between the two reviewers.

A limitation of our review is that we were unable to explore associations between the use of behaviour change techniques and change in health behaviour or other health-related outcomes. This is because none of the apps have been systematically evaluated and highlights the need for well-designed studies to determine the effectiveness of health and wellness apps against a range of process and health-related outcomes.

A further limitation is that we only reviewed top-rated apps in the two most popular app stores and did not sample the entire range of apps available. Thus, the range of health behaviours targeted will reflect the preferences of the consumers rather than covering the entire repertoire of apps offered by developers. It is possible that apps with certain characteristics, for example, high behaviour change content, are less popular with users and we were not able to test this hypothesis. Nevertheless, we were able to study the use of behaviour change techniques in apps in common use, which was the objective of our study.

In this review, we focused on commonly used behaviour change techniques. It would be interesting to examine behaviour change techniques that were not used or had a low frequency of use, to determine how these aligned with relevant behavioural and cognitive theories and hence identify any potential opportunities for app developers. Similarly, we did not examine the frequency with which behaviour change techniques were used in each individual app and the mode of delivery of each behaviour change technique. Future work in larger data sets might usefully make these more detailed observations and could also examine the effects of prespecified, theoretically consistent groups of behaviour change techniques against relevant outcomes.

### Implications for clinicians and policymakers

Smartphone games could provide a potentially cost-effective platform for health promotion and, thus, could have a substantial public health impact. An efficient mechanism will be needed to promote those apps that are most likely to bring health benefits. Only three apps in our review were approved by the NHS Health Apps Library, which is intended to provide this function for consumers in the UK. While this may be because other apps were reviewed and not approved, it is possible that the Library in its current form does not present the full range of apps available to the public. The NHS Library is currently updating review processes aiming to provide an accredited set of apps, which have been endorsed and given a service quality certification mark by The British Standards Institution (Kitemark) through NHS Choices.^54^

The majority of apps that we identified focused on exercise and fitness. There were very few gamified apps targeting health behaviours more directly relevant to clinical outcomes, highlighting a potential gap in the market and possible untapped resource for health promotion. It is possible that the task of encouraging exercise and fitness lends itself more easily to gamification and that application of gamification to other aspects of health promotion will be more challenging. However, another explanation may be that health and fitness apps are simply more popular since we searched only the top-rated apps in the most popular stores. In the latter case, the challenge will be to make apps and smartphone games that are as appealing to users as those promoting exercise and fitness.

### Unanswered questions and future research

This review provides evidence to inform further research in the growing field of gamification in healthcare apps and to determine optimum use of behaviour change constructs in smartphone games. The relationship between the behaviour change technique content of an intervention and the resulting health behaviour change is not simple.^34^ ^35^ ^37^ More techniques are not necessarily better and further work is needed on the specific combinations of techniques likely to be effective in smartphone games.

There may be potential for more effective apps to be developed drawing from the full repertoire of techniques and combinations of techniques, which are appropriate to this platform. This development will require multidisciplinary collaboration between game developers, behaviour change experts and public health specialists.

Further research and clinical evaluation is urgently needed for healthcare apps to assess their effectiveness in modifying health behaviour and the clinical consequences of these behaviour changes. None of the apps in our review has been evaluated in randomised controlled trials to quantify potential benefit and harms that may arise from use of this technology. There is a need for regulation of healthcare apps and strengthened approval mechanisms to ensure patients have access to effective and safe interventions. The British Standards Institution has formulated and published a code of practice for health and wellness apps, providing app developers with quality criteria to consider during the development process.^55^ We suggest that this code should be widely adopted and could lead to better quality and more effective products.

The economics of production and scale of delivery could potentially give smartphone apps an advantage over other health promotion interventions. Similar methods of assessing cost-effectiveness could be used as for other health technologies (https://www.nice.org.uk/about/what-we-do/our-programmes/nice-guidance/nice-medical-technologies-guidance).

## Conclusions

We provide an overview of the use of behaviour change techniques in the rapidly developing area of smartphone games, aiming to provide insights to inform more effective development of applications to change health-related behaviours. We suggest that strengthening collaboration between app developers, behavioural scientists and public health practitioners is necessary to realise the full health benefits of this new technology, which could be substantial. The benefits and harms arising should be evaluated using standard methods to enable consumers to make appropriate choices and allow health systems to make decisions about reimbursement.
