# Supplementary material for: Gamification for health promotion: systematic review of behaviour change techniques in smartphone apps
Source: BMJ Open. 2016 Oct 4;6(10):e012447. doi: 10.1136/bmjopen-2016-012447 (PMC5073629; doi:10.1136/bmjopen-2016-012447)
Supplement: supplementary prospero file [file bmjopen-2016-012447supp_prospero_file.pdf]

PROSPERO International prospective register of systematic reviews

‘Gamification’ for health behaviour change in smartphone apps: systematic review of behaviour change techniques

Elizabeth Edwards, Jim Lumsden, Carol Rivas, Liz Steed, Lindsey Edwards, Arun Rajan, Ratna Sohanpal, Hope Caton, Steph Taylor, Robert Walton

Citation

Elizabeth Edwards, Jim Lumsden, Carol Rivas, Liz Steed, Lindsey Edwards, Arun Rajan, Ratna Sohanpal, Hope Caton, Steph Taylor, Robert Walton. ‘Gamification’ for health behaviour change in smartphone apps: systematic review of behaviour change techniques. PROSPERO 2015:CRD42015029841 Available from
http://www.crd.york.ac.uk/PROSPERO_REBRANDING/display_record.asp?ID=CRD42015029841

Review question(s)

What proportion of Health Care apps on the official Apple and Android stores use gamification to modify health behaviour?

What behaviour change techniques and combinations of techniques are used?

Is there an association between behaviour change techniques and combinations of techniques with NHS library ratings, user ratings and price?

Searches

We aim to identify all English language health apps using gamification and available on the official Apple and Android app stores that are ‘top rated’, defined by Apple and Google Play stores (https://play.google.com/store)(https://itunes.apple.com).

The following search terms will be used: rewards, prizes, avatar use, badges, leaderboards, competitions, health-related challenges. All apps meeting the inclusion criteria will be recorded in a standardized structured form.

Types of study to be included

Not applicable

Condition or domain being studied

We plan to review all apps that aim to modify any health behaviour.

Participants/ population

Inclusion criteria:

English language apps

Apps that use the following gamification techniques: rewards, prizes, avatar use, badges, leader boards, competitions, health-related challenges

Apps available through google play and I Tunes

Smart phone app

Apps in the Medical, Health & Wellness or Health & Fitness section

Apps targeted at users of any age

Free and paid apps

Exclusion criteria:

Apps designed for tablets

Non- English language apps

Non-health related apps

Apps that do not contain gamification techniques

Apps designed for Health Care professionals

Intervention(s), exposure(s)

The identified apps will be screened independently by two review authors (EE, JL) trained in coding behaviour change techniques. Coding will be carried out independently by the two reviewers who will then cross-check for discrepancies. Responses from each review author will be recorded independently in a standardized structured form.

App content will be assessed against criteria derived from a behaviour change taxonomy used in health behaviour change intervention established by Michie et.al [1]. The taxonomy consists of 16 categories and 93 individual techniques. The number of individual techniques included in each app will be counted (range 0-93). The use of behaviour change categories, individual techniques and combinations of techniques will be examined. Associations with NHS library ratings, user ratings and price will also be explored.

References:

1. Michie, S., et al., The behavior change technique taxonomy (v1) of 93 hierarchically clustered techniques: building an international consensus for the reporting of behavior change interventions. Ann Behav Med, 2013. 46(1): p. 81-95.

Comparator(s)/ control

Not applicable

Outcome(s)

Primary outcomes

The number of Health Care apps to incorporate gamification:

We will assess the proportion of apps in the official Apple and Google Play stores and NHS health apps library to incorporate gamification in order to modify health behaviour. A systematic search of the top rated free and paid Medical, Health & Wellness, Health & Fitness apps will be conducted.

The use of behaviour change techniques and combination of Behaviour change technique:

The number of individual techniques included in each app will be counted (range 0-93). The mean number, median value and the range will also be calculated. We will investigate what behaviour change categories, individual techniques and combinations of techniques are used.

The association of behavior change technique use with downloads, user ratings, price and NHS library ratings:

We will explore associations between number of individual behaviour change technique and combination of behaviour change techniques with downloads, user ratings, price and NHS library ratings. Customer ratings will be obtained from the Apple store and Google Play. The total rating for all versions of the app will be used. The price will be recorded as free or paid. The exact price of each app will also be recorded.

As above, under each Primary outcome

Secondary outcomes

Not applicable

Data extraction, (selection and coding)

The initial systematic review of top rated Medical, Health & Wellness, Health & Fitness apps on the official Apple and Google Play stores and NHS health apps library will be conducted by one review author (EE). Assessments against eligibility criteria will be recorded on a standardized structured form.

Medical, Health & Wellness or Health & Fitness apps meeting inclusion criteria will be downloaded on to a test device. The same test device will be used throughout the evaluation. Test devices will be unmodified consumer-grade smartphones running up-to-date versions of their mobile operating system. The same version of each app will be used throughout testing.

The identified apps will then be screened independently by two review authors (EE, JL) trained in BCT coding. Any discrepancies will be discussed with a third review author (LS). Responses from each review author will be recorded independently in a second standardized structured form.

Extracted information will include: version, date of first release, date of latest update, publisher, description of the app, main function of the app, target user, special features incorporated, number of downloads in the Android store, number in the chart in the Apple store. Missing data will be requested from the author/publisher or the app or Apple/Android store.

Risk of bias (quality) assessment

Not Applicable

Strategy for data synthesis

A quantitative synthesis of the number of apps to contain gamification will be conducted including all apps that meet the inclusion criteria and that are identified in the review. Basic descriptive statistics will be calculated. Apps found in both the Apple store and Google Play store will not be included twice and will be recorded only in the Apple iPhone data. The number of Behaviour change technique and combination of Behaviour change technique will be compared to user ratings, price and NHS library ratings. Apps that do not have customer ratings available will not be included in this analysis. Statistical significance of the association between the number of behaviour change techniques and the combination of behaviour change techniques with user ratings, price and NHS library ratings will be determined by linear regression or Spearman’s Rank Correlation Coefficient as appropriate. This will be calculated using GraphPad Prism 6.

Analysis of subgroups or subsets

None Planned

Dissemination plans

The results of this research will be disseminated in a number of ways to all stakeholders. Professional groups both in the Academic/Clinical setting and Technologists will be informed through presentation at local, National and International conferences/meetings. Results of the review will be published in highly cited and open access peer reviewed journals. Relevant public health, NHS and other healthcare organisations including policy and decision makers will be informed of the results of the research. This will be at both local and national meetings.

Contact details for further information

Dr Edwards

Barts and The London School of Medicine and Dentistry

Queen Mary University of London

Centre for Primary Care and Public Health, Blizard Institute

Yvonne Carter Building

58 Turner Street

London

E12AB

elizabethedwards@doctors.org.uk

Organisational affiliation of the review
Barts and The London School of Medicine and Dentistry, Queen Mary University of London, Centre for Primary Care and Public Health, Blizard Institute

http://blizard.qmul.ac.uk/centres/centre-for-primary-care-and-public-health.html

Review team
Dr Elizabeth Edwards, Centre for Primary Care and Public Health, Bart's and The London School of Medicine and Dentistry
Mr Jim Lumsden, School of Experimental Psychology University of Bristol
Dr Carol Rivas, Faculty of Health Sciences, University of Southampton
Dr Liz Steed, Centre for Primary Care and Public Health, Bart's and The London School of Medicine and Dentistry
Dr Lindsey Edwards, MRC Centre for transplantation, Kings College London
Dr Arun Rajan, Centre for Primary Care and Public Health, Bart's and The London School of Medicine and Dentistry
Dr Ratna Sohanpal, Centre for Primary Care and Public Health, Bart's and The London School of Medicine and Dentistry
Ms Hope Caton, Department of Computing and Information Systems, Kingston University
Professor Steph Taylor, Centre for Primary Care and Public Health, Bart's and The London School of Medicine and Dentistry
Professor Robert Walton, Centre for Primary Care and Public Health, Bart's and The London School of Medicine and Dentistry

Anticipated or actual start date
01 December 2014

Anticipated completion date
01 January 2016

Funding sources/sponsors
Supported by the NIHR Programme grant RP-PG-0609-10181

Conflicts of interest
None known

Language
English

Country
England

Subject index terms status
Subject indexing assigned by CRD

Subject index terms
Behavior Therapy; Health Behavior; Humans; Smartphone

Stage of review
Ongoing

Date of registration in PROSPERO
02 December 2015

Date of publication of this revision
02 December 2015

**Stage of review at time of this submission**	**Started**	**Completed**
Preliminary searches	No	Yes
Piloting of the study selection process	No	Yes
Formal screening of search results against eligibility criteria	No	Yes
Data extraction	No	No
Risk of bias (quality) assessment	No	No
Data analysis	No	No

PROSPERO
International prospective register of systematic reviews
The information in this record has been provided by the named contact for this review. CRD has accepted this information in good faith and registered the review in PROSPERO. CRD bears no responsibility or liability for the content of this registration record, any associated files or external websites.
