# Supplementary material for: Gamification for health promotion: systematic review of behaviour change techniques in smartphone apps
Source: BMJ Open. 2016 Oct 4;6(10):e012447. doi: 10.1136/bmjopen-2016-012447 (PMC5073629; doi:10.1136/bmjopen-2016-012447)
Supplement: supplementary figures [file bmjopen-2016-012447supp_figures.pdf]

**Supplementary Figures**

Supplementary Figure 1: Example of app content coding with individual behaviour change techniques

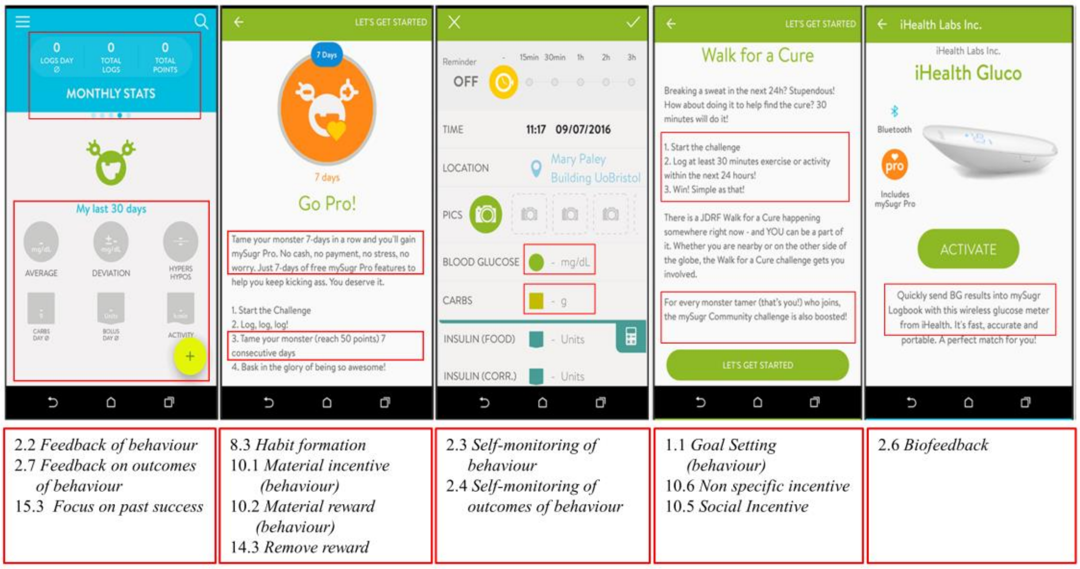


An example of behaviour change technique coding using the standard 93 category taxonomy[7]. Screen-shots of the 'DiabetesCompanion' by mySugr[52] are shown with examples of behaviour change techniques highlighted. Of note, some screen shots may have multiple behaviour change techniques, which have not been highlighted but would have been coded accordingly.

Supplementary Figure 2: Number of behaviour change techniques per app

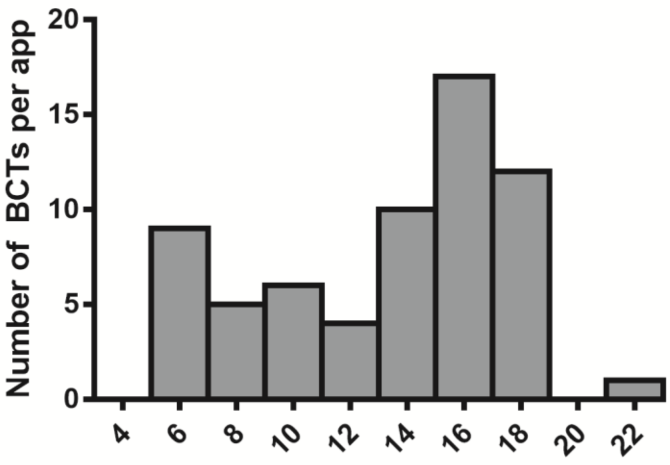


Histogram of the number of behaviour change techniques included per app.
