# Supplementary material for: Gamification for health promotion: systematic review of behaviour change techniques in smartphone apps
Source: BMJ Open. 2016 Oct 4;6(10):e012447. doi: 10.1136/bmjopen-2016-012447 (PMC5073629; doi:10.1136/bmjopen-2016-012447)
Supplement: supplementary table — Characteristics of included apps [file bmjopen-2016-012447supp_table.pdf]

Supplementary Table 1: Characteristics of included apps

Name of App	Category	Source	Targeted behaviour	Cost	User ratings	Number of BCTs
My Diet Coach – Pro	Health & Wellness	Android	Weight loss	£1.99	4.5	22
Nike+ Running	Health & Fitness	Apple iPhone	Exercise	£0.0	4	18
Speedo Fit	Health & Fitness	Apple iPhone	Exercise	£0.0	3.5	18
Runtastic Sit Ups Trainer PRO	Health & Fitness	Apple iPhone	Exercise	£1.49	4.5	18
Runtastic Squats Trainer	Health & Fitness	Apple iPhone	Exercise	£0.0	4.5	18
Diabetes Companion by mySugr	Medical	Apple iPhone	Blood sugar monitoring adherence	£0.0	5	18
Runtastic Squats PRO	Health & Wellness	Android	Exercise	£1.91	4.7	18
Runtastic Pull-Ups PRO	Health & Wellness	Android	Exercise	£1.73	4.5	18
Runtastic Sit-Ups	Health & Wellness	Android	Exercise	£0.0	4.4	18
Runtastic Push-Ups	Health & Wellness	Android	Exercise	£0.0	4.5	18
Fitocracy	Health & Fitness	Apple iPhone	Fitness	£0.0	5	17
UP	Health & Fitness	Apple iPhone	Fitness	£0.0	2.5	17
RunKeeper - GPS Track Run Walk	Health & Wellness	Android	Exercise	£0.0	4.4	17
Run with Map My Run	Health & Fitness	Apple iPhone	Exercise	£0.0	5	16
Map My Ride+ GPS Cycling Riding	Health & Fitness	Apple iPhone	Exercise	£1.99	4.5	16
Map My Ride - GPS Cycling, Riding, Mountain Biking, and Workout Tracking	Health & Fitness	Apple iPhone	Exercise	£0.0	4.5	16
Map My Fitness	Health & Fitness	Apple iPhone	Fitness	£0.0	4.5	16
Adrian James 6 Pack Abs Workout Lite	Health & Fitness	Apple iPhone	Exercise	£0.0	5	16
Run with Map My Run+	Health & Fitness	Apple iPhone	Exercise	£1.99	4.5	16
Zombies, Run!	Health & Fitness	Apple iPhone	Exercise	£2.49	4.5	16
Map My Walk+ GPS Pedometer	Health & Wellness	Android	Exercise	£1.91	4.4	16
Map My Run+ GPS Running	Health & Wellness	Android	Exercise	£1.99	4.5	16
Map My Fitness+ Workout Trainer	Health & Wellness	Android	Exercise	£0.69	4.4	16

Map My Hike+ GPS Hiking	Health & Wellness	Android	Exercise	£0.77	4.4	16
Quit-Smoking Coach	Health & Wellness	Android	Smoking Cessation	£2.90	4.4	16
STOP Cigarettes PRO Quit Smoking	Health & Wellness	Android	Smoking Cessation	£1.86	4.8	16
Map My Walk GPS Walking	Health & Wellness	Android	Exercise	£0.0	4.4	16
Map My Run GPS Running	Health & Wellness	Android	Exercise	£0.0	4.5	16
Endomondo Sports Tracker	Health & Fitness	Apple iPhone	Exercise	£0.0	3	15
Nike+ FuelBand	Health & Fitness	Apple iPhone	Exercise	£0.0	4	15
Strava Cycling	Health & Fitness	Apple iPhone	Exercise	£0.0	5	14
5K runner free	Health & Fitness	Apple iPhone	Exercise	£0.0	4.5	14
Strava Run	Health & Fitness	Apple iPhone	Exercise	£0.0	5	14
5K Runner paid	Health & Fitness	Apple iPhone	Exercise	£1.99	5	14
Zombies, Run! 5k Training Zombies	Health & Fitness	Apple iPhone	Exercise	£1.49	4.5	14
Freeletics	Health & Wellness	Android	Fitness	£1.99	4	14
Fitbit	Health & Fitness	Apple iPhone	Fitness	£0.0	4	13
JEFIT Pro - Workout & Fitness	Health & Wellness	Android	Exercise	£2.99	4.6	13
Virtuagym Fitness Home & Gym	Health & Wellness	Android	Exercise	£0.0	4.2	13
100% Army Fit	Health & Wellness	Android	Fitness	£0.0	4.4	13
Karrimor Elite	Health & Wellness	Android	Exercise	£0.0	4.4	12
7 Minute Workout “Seven” with High Intensity Interval Training Challenge	Health & Fitness	Apple iPhone	Exercise	£0.0	4.5	11
Change For Life- Smart Restart	Health & Fitness	Apple iPhone	Fitness	£0.0	4.5	11
Total Fitness - Gym & Workouts	Health & Wellness	Android	Exercise	£0.0	4.4	11
Aquafresh Brush Time	Health & Fitness	Apple iPhone	Tooth brushing	£0.0	3.5	10
Adidas Snapshot	Health & Fitness	Apple iPhone	Exercise	£0.0	4.5	10
Stop Smoking Pro	Health & Wellness	Android	Smoking cessation	£1.99	4.3	10
RunTroll	Health & Wellness	Android	Exercise	£0.0	4.9	10
Steps Mania Pro	Health & Wellness	Android	Exercise	£0.62	4.2	9

Stop Smoking	Health & Wellness	Android	Smoking cessation	£0.0	4.1	9
MyFit Fitness	Health & Fitness	Apple iPhone	Fitness	£0.0	3.5	8
The Spartan 300 Workout	Health & Wellness	Android	Exercise	£3.10	4.3	8
Disney Magic Timer by Oral-B	Health & Wellness	Android	Tooth brushing	£0.0	3	7
Runtastic Road Bike	Health & Wellness	Android	Exercise	£0.0	4.6	7
Plank Challenge	Health & Wellness	Android	Exercise	£0.0	4	7
Runmeter GPS Pedometer	Health & Fitness	Apple iPhone	Exercise	£0.0	5	6
Cyclemeter GPS	Health & Fitness	Apple iPhone	Exercise	£0.0	5	6
Walkmeter GPS Pedometer	Health & Fitness	Apple iPhone	Exercise	£0.0	4.5	6
Gorilla Workout: Strength Plan	Health & Wellness	Android	Exercise	£0.62	4.3	6
Fitness Flow	Health & Wellness	Android	Fitness	£2.75	4.5	6
AllSport GPS PRO	Health & Wellness	Android	Exercise	£3.10	4.1	6
Fitness Flow FREE	Health & Wellness	Android	Adult Fitness	£0.0	4	6
Change4Life fun generator	Health & Fitness	Apple iPhone	Fitness	£0.0	3	5
Runtastic Pedometer	Health & Wellness	Android	Exercise	£0.0	4.3	5

Supplementary Table 1: Characteristics of included apps

Characteristics of apps meeting the inclusion criteria including: name of app, given category in the app store, source of app identified as either being from the Apple or Android store, targeted behaviour, cost of the app in sterling, user ratings as defined by either Apple or Android stores and number of individual behaviour change techniques included.
